# Effectiveness of digital health using the transtheoretical model to prevent or delay type 2 diabetes in impaired glucose tolerance patients: protocol for a randomized control trial

**DOI:** 10.1186/s12889-019-7921-8

**Published:** 2019-11-21

**Authors:** Rasmieh Alzeidan, Zeinab Shata, Marwah Mazen Hassounah, Leena Rashad Baghdadi, Ahmad Hersi, Amel Fayed, Tarek Kashour, Hala Elmorshedy

**Affiliations:** 1 Cardiac Sciences Department, College of Medicine, King Saud University, Riyadh, Saudi Arabia; 2 High Institute of Public Health, Alexandria University, Alexandria, Egypt; 3 Community Medicine Unit, Family and Community Medicine Department, College of Medicine, King Saud University, Riyadh, Saudi Arabia; 4 Department of Family and Community Medicine, King Saud University and King Khalid University Hospital, Riyadh, Saudi Arabia; 5 Princess Nourah Bint Abdulrahman University, College of Medicine, Riyadh, Saudi Arabia

**Keywords:** Transtheoretical model, Type 2 diabetes mellitus, Glycated hemoglobin, Cardiovascular diseases, Healthy lifestyle, Diet, Physical activity

## Abstract

**Background:**

There is high prevalence of prediabetes and type 2 diabetes mellitus (T2DM) in Saudi Arabia that is still increasing. Early diagnosis of prediabetes, and immediate, effective intervention is yet unestablished. Conventional health promotion approaches are used to educate prediabetic patients. Behavior modification is very effective in prediabetics to delay T2DM. Thus, the main objective of this study is to examine the effect of the new behavioral model, the Transtheoretical Model short messages (text 4 change) to modify lifestyle to prevent or delay the onset of T2DM, through promotion of a healthy diet and increased physical activity, in impaired glucose tolerance patients. Another objective is to estimate the impact of this model on markers of cardiovascular and metabolic risks as T2DM is one of the modifiable risk factors to prevent cardiovascular diseases.

**Methods:**

This is a randomized controlled trial. One thousand and sixteen, eligible Saudi adults will be recruited from the Heart Health Promotion study (HHP), which was conducted at the King Saud University from July 2013 to April 2014. These adults were at a higher risk of developing T2DM within 2–3 years. The research team’s database has a contact list and they will recruit individuals over 6–8 weeks. All participants will be randomized at a 1:1 ratio into two groups, receive group education about lifestyle modifications and written information about diet and physical activity. Text 4 change SMS texts will be sent only to the intervention group. All participants will be assessed at baseline, 6, 12, 18, 24, 30, and 36 months for behavioral change using a World Health Organization (WHO) STEPS questionnaire and for glycated hemoglobin, biochemical and anthropometric measurements using standard methods.

**Discussion:**

This new approach for promoting the importance of behavior modification in prediabetics is expected to delay and/or prevent the development of T2DM in Saudi Arabia, subsequently reducing the risk of cardiovascular morbidity and mortality too. Results from this study will promote an innovative and high-tech way to decrease the burden of cardiovascular diseases in Saudi Arabia.

**Trial registration:**

International Standard Randomized Control Trial, registration number ISRCTN10857643. Registered 4 June, 2018.

## Background

Impaired glucose tolerance (IGT) and impaired fasting glucose (IFG) are intermediate conditions sometimes called prediabetes, where blood glucose levels are above normal but below diabetic thresholds [1]. Seventy percent of individuals with prediabetes eventually develop type 2 diabetes mellitus (T2DM) [2]. While people with IFG have a 20–30% chance of developing diabetes over the course of 5–10 years [3–5], this chance escalates to 90% if the individual is diagnosed with IGT [6]. In Saudi Arabia, the prevalence of both prediabetes and T2DM is high (18.3 and 11.9%, respectively) and increases with age [7]. Even though T2DM is treatable, it is usually associated with other comorbidities such as hypertension, hypercholesterolemia and obesity. These conditions in patients with T2DM contribute to the development of cardiovascular diseases (CVD). Thus, T2DM is one of the modifiable risk factors that can decrease the risk of several chronic diseases such as CVD, and reduce its associated mortalities. A population-based study from England showed that prediabetes may convert back to normal glycemia [8]. Furthermore, landmark clinical trials have proven the effectiveness of lifestyle interventions towards a healthy diet and sufficient physical activity to reduce or delay the occurrence of T2DM by 50% in prediabetic individuals [9–11]. The Center For Disease Control and Prevention (CDC), and the American College of Sports Medicine have demonstrated that accumulation of 10,000 steps per day on most days of the week is an important component of improving glycemic control, insulin sensitivity, and/or cardiovascular (CV) risk in patients with T2DM [1, 12].

Targeting this at risk population of prediabetics and increasing their awareness about the importance of a healthy lifestyle will help in the long run, to significantly decrease the prevalence of T2DM in Saudi Arabia. While many educational programs have been used to promote health in the Saudi community, the use of a digital approach is still in its early stages [7]. Nowadays, the daily use of smartphones is vital due to their efficiency, connectivity and functionality. This makes the delivery of educational messages easier and more convenient.

Prediabetes complications have been associated with the long-term burden of CVD, which is a major cause of death among diabetic patients [13, 14]. The risk of developing CVD increased 2–4 fold compared to age-sex matched patients without diabetes [15]. Worldwide, 43% of diabetes patients are more likely to die prematurely, and the rate of these deaths was higher in developing countries compared to developed countries [16]. Diabetes has a negative impact on the individuals’ productivity and quality of life [17]. Diabetes also places a heavy economic burden on individuals, families, societies and countries. For example, globally, in 2017 diabetes consumed USD 727 billion from the health expenditure. In 2014, 14% of the total health expenditure of Saudi Arabia was utilized for diabetes management [18]. Recent studies showed that the prevalence of prediabetes among the general population in Saudi Arabia was 25.5% in 2014 [19], and 27% among university employees and their families in 2016 [20].

The 2018–2020 National Transformation Program delivery plan, one of the Kingdom’s Vision 2030 programs, aims to improve health outcomes through e-Health. Over the last decade, the mobile phone short message service (SMS) has been increasingly used to deliver interventions designed to enhance healthy behavior [21]. SMS messages have the advantage of being inexpensive, convenient, reach the intended person instantly, and can be tailored to the individual [22]. In Saudi Arabia, the mobile phone market witnessed the fastest growth in the Middle East region with 23 million smartphones in use in 2017 [23].

Theory-led interventions base components of a health intervention on behavioral change, this theory has been constructed and called ‘the Transtheoretical Model (TTM)’ [24]. TTM has been used to improve adherence to medications in patients with CV risk factors [25, 26], and to prevent or delay the onset of T2DM in people with IGT [27, 28]. It has also been used effectively to predict or measure behavioral change in eating habits [29] and physical activity [30].

TTM posits that behavior change passes through five stages, which are precontemplation, contemplation, preparation, action, and maintenance (Table 1**)**. These stages indicate the individuals’ willingness to change their behavior ranging from long-term inactive (i.e. precontemplation stage) to long-term active (i.e. maintenance) behavior. Each stage has processes of change that are methods to move an individual from one stage of behavior to the other (Table 2) [24]. For example, raising consciousness (a process of change) is more effective with an individual in the precontemplation and contemplation stages rather than a person in the maintenance stage. Consequently, tailoring interventional messages based on the individual’s stage of change would be more effective in progressing them to the next stage of change [32].

**Table 1:** Stages of change with brief descriptions

Stage of change	Description
Precontemplation (PC)	The individual is not willing to change in the foreseeable future (measured as the next 6 months). Individuals in this stage are mostly uninformed or demoralized.
Contemplation (C)	The individual is willing to change in the next 6 months. Individuals in this stage are aware of some pros of behavior change but are still more inclined to value the cons.
Preparation (P)	The individual is willing to change in the foreseeable future (measured as the next month) and has already taken some small steps towards change (in the past year). Individuals in this stage usually have some plan on how to tackle this inactiveness.
Action (A)	The individual has changed, but not longer than 6 months. Individuals in this stage have ‘changed’ but have not reached the duration which exemplifies real behavior change.
Maintenance (M)	The individual has changed, longer than 6 months. Individuals in this stage have changed and are working not to relapse.
Adapted from open access of reference [31]

**Table 2:** Processes of change in terms of experimental and behavioral processes (a brief description)

Process	Description	
Experiential processes		
Consciousness raising (CR)	The individual seeks increased knowledge about the causes, consequences and cures for their problem behavior.	From precontemplation to contemplation
Dramatic relief (DR)	The individual’s emotions about the problem behavior and possible solutions are evoked.	
Environmental reevaluation (ER)	The impact that the individual’s problem behavior has on their environment is reevaluated.	
Social liberation (SOL)	Attempts are made to increase alternatives for the individual’s former problem behavior.	
Self-reevaluation (SR)	Cognitions and emotions regarding the individual with respect to their problem behavior are reevaluated.	From contemplation to preparation and action
Behavioral processes		
Self-liberation (SEL)	The individual has the belief that he can change and commits to it by choosing a course of action.	
Helping relationships (HR)	The individual seeks trust and open discussion about the problem behavior as well as support for the healthy behavior change.	Action to maintenance
Counter-conditioning (CC)	The individual substitutes positive behaviors for the individual’s problem behavior.	
Reinforcement management (RM)	Steps or changes made by the individual are rewarded when in a positive direction or punished when in a negative direction.	
Stimulus control (SC)	Stimuli that may cue a lapse back to the problem behavior are avoided and prompts for healthier alternatives are inserted.	
Adapted from reference [31]		

Therefore, this study aims to examine the effect of an interventional TTM based on short messages (text 4 change) of lifestyle modification to prevent or delay the onset of T2DM. The intervention will promote a healthy diet and increased physical activity among subjects with IGT. The design for this trial is parallel groups, the allocation ratio will be 1:1, and superiority framework.

### Primary objective

To examine the effect of the TTM short messages (text 4 change) to modify lifestyle to prevent or delay the onset of T2DM, through promoting a healthy diet and increased physical activity among subjects with IGT.

### Secondary objectives

To estimate the impact of the TTM-based SMS messages (text 4 change) on regulating the level of biomarkers for cardiovascular and metabolic risks, lowering the indices of general and central obesity, and maintaining body measurements among nonobese individuals. To estimate the impact of the intervention program on the incidence of myocardial infarction, stroke, death and hospitalization. To examine the acceptability of the text 4 change motivational SMS messages among the intervention group.

## Methods/design

### Study design (setting, eligibility and recruitment)

This is a three-year randomized controlled trial that includes Saudis ≥18 years of age. Participants for the present study will be recruited from the Heart Health Promotion study (HHP), which was conducted at King Saud University from 8 July, 2013 to 30 April, 2014 [20]. In the HHP study, participants were recruited from the three primary health care clinics serving the employees and their families at King Khalid University Hospital [20]. The HHP study was conducted on a large group of Saudi adults, who were at a higher risk of developing T2DM within 2–3 years. Recent data record updates of the original study on May 2017, revealed that out of the total sample (1234 prediabetic subjects) enrolled in the HHP study, 218 individuals had developed overt diabetes during the last 4 years (May 2013–May 2017). Therefore, 1016 are eligible for the present study.

### Inclusion criteria

The inclusion criteria for participants is Saudi adults aged ≥18 years, literate, prediabetic, with no contraindication of physical activity, have a smartphone with easy access to the internet and are willing and able to provide written informed consent to participate in the study.

### Exclusion criteria

The exclusion criteria for participants is pregnancy, individuals who do physical exercise > 50 min/week, follow a diet regimen, have a history of myocardial infarction, have impaired renal function, and are diabetic.

### Recruitment

**Fig. 1:**
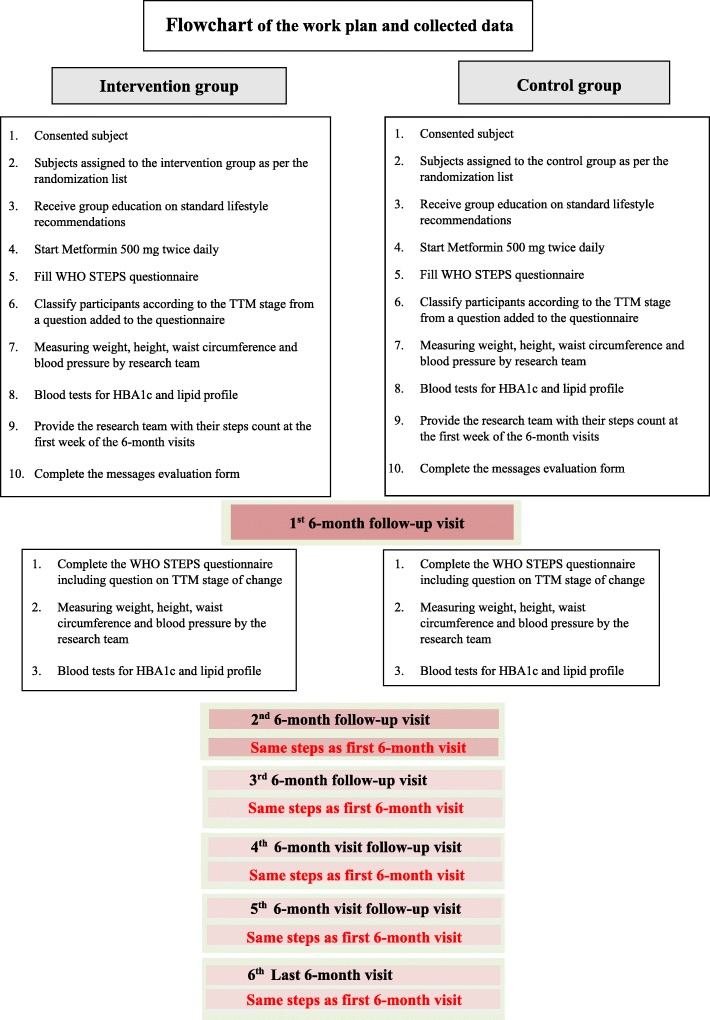
Flowchart for the work plan and data collection

Recruitment will take place over a 2-month period in order to ensure that all eligible subjects (from the HHP study) who fulfil the inclusion criteria are invited, give consent, and are enrolled following the steps of the baseline visits as shown in the flowchart (Fig. 1). The research team’s database has a contact list and they will recruit individuals over 6–8 weeks. Subsequently, they will be given a 6-month follow-up visit appointment.

### Randomization and blinding

Recruited, eligible participants will be randomized into two groups (1:1 ratio), using random numbers table generated in the Statistical Package for Social Studies (SPSS v. 21) (SPSS v. 21, IBM Corp., New York, NY, USA) by an independent statistician. The laboratory staff, biostatistician, health educator, and principal investigator will be blinded to the participant’s group allocation until the end of the study. The participants will not be blinded to the intervention due to its nature, as well as the research assistant/project manager who is responsible for disseminating the text message intervention.

### Baseline visit

#### Lifestyle education

Eligible prediabetic subjects from the previous HHP study [20] will be invited to the HHP office. If they give consent for this trial, then participants will be assigned to either the intervention or the control group by the computer-generated list. At this baseline visit, all participants will receive group education and motivation about lifestyle modifications along with written information about diet and physical activity recommendations offered by a diabetic educator and a physician. This will advise participants on balancing food intake and physical activity to achieve and maintain appropriate body weight. All participants will take Metformin 500 mg twice daily. In addition, only participants in the intervention group will receive SMS messages at frequent intervals. These messages will be about a healthy lifestyle, the benefits of physical activity and a healthy diet. Neither group will receive any additional lifestyle information or advice by personal contact after the baseline visit, except in response to specific queries from the participants.

#### Physical measurements

At the baseline visit, all recruited participants’ weight, height, waist circumference, and blood pressure from the left arm will be measured in a sitting position, and the mean of two readings will be considered. Blood tests for HbA1c and lipid profiles will be performed by qualified laboratory personnel. Glycated hemoglobin (HbA1c) and fasting lipid profiles will be measured for all participants at baseline and at every 6-month follow-up visit.

#### Assessment of interventions

All participants will complete the WHO STEPS instrument version 3.1 at the baseline visit and during each follow-up visit. This will assess behavioral risk factors including healthy diet, physical activity and the participants’ behavioral change(s) stage based on the TTM at the baseline visit and follow-up visits for progression in the stages of change.

Data about adherence to physical activity and healthy dietary intake recommendations will be recorded at every 6-monthly follow-up visit. Adherence will be self-reported every 6 months and scored as poor, moderate or good. Physical activity and dietary intake will be categorized as adherent or nonadherent for statistical analysis.

At every visit after the baseline visit, we will assess the mobile messages in the intervention group through a specially designed questionnaire. It will assess the user acceptability and satisfaction of the SMS messages program using a scoring system of 0–10. It will assess message content, frequency, clarity, effect on personal life (“degree of disturbance”), and personal views of the impact of these messages on lifestyle. The list of questions also includes the preferred time to receive messages and invites suggestions for improvement. A maximum total score of 10 denotes that the text messages were highly acceptable and satisfactory, while the lowest score of zero denotes that the text messages were the least acceptable and unsatisfactory.

#### Six-monthly follow-up visits

Subjects will be given appointments for follow-up visits at the baseline visit, 6, 12, 18, 24 and 30 months, a total of six visits plus the baseline visit. Therefore, all subjects will repeat steps 1–3 of the first 6-month follow-up visit, at each of these visits as shown in the flowchart (Fig. 1).

### Definitions and measurements

#### Definitions of cardiometabolic risk factors

##### **Prediabetes** [Impaired glucose tolerance (IGT) and impaired fasting glucose (IFG) [33, 34]]

Subjects will be defined as prediabetic, if the level of glycated hemoglobin (HbA1c) is 5.7–6.4% as per WHO and American Diabetes Association (ADA) criteria [35], or the subject was previously diagnosed as prediabetic.

### Diabetes mellitus

Diabetes mellitus will be defined as per WHO and ADA criteria of HbA1c level ≥ 6.5% or the subject being previously diagnosed as diabetic and using anti-diabetes medication [35].

### Dyslipidemia

Subjects will be categorized as having any sort of dyslipidemia according to the WHO and the Third Adult Treatment Panel (ATP-III) of the National Cholesterol Education Program (NCEP). Dyslipidemia includes raised levels of total cholesterol, and/or low-density lipoprotein cholesterol (LDL-C), and/or triglycerides and low levels of high-density lipoprotein cholesterol (HDL-C), or if the subject reported using medications to lower blood lipid levels [36, 37].

### Metabolic syndrome (MetS)

Participants will be identified as having MetS using the NCEP-ATPIII criteria, if the subject has at least three out of five factors including abdominal obesity, dyslipidemia (raised triglycerides and reduced HDL-C), raised fasting plasma glucose and hypertension [38].

### Physical activity

Participants will be identified as physically active if they achieve 150 min per week (at least 5 days per week) of moderate activity such as brisk walking or 1 hour of vigorous activity [39]. Furthermore, in the current study, we will measure their achieved steps per week, considered in the 1st week of each 6-month follow-up visit using a pedometer through health applications in the smartphones to count steps.

### Measurements of physical activity adherence

Physical activity adherence was classified into three. Poor**:** Less than 150 min/week of moderate activity or 1 hour of vigorous activity (non-adherent). Fair: About 150–250 min/week of moderate activity or 1 h, 130 min of vigorous activity (adherent).

Good: More than 250 min/week of moderate activity, or if work involved, vigorous work (adherent).

### Healthy eating

Healthy eating such as following the food pyramid and encouraging participants to consume the recommended portions of fruit and vegetables and avoiding unhealthy dietary habits, according to the WHO recommendations for a healthy diet [40]. This includes avoiding fast food, fatty food, sugar-sweetened beverages and refined carbohydrates, decreasing salt intake (< 5 g/day equivalent to sodium intake of < 2 g per day), encouraging more fiber-rich food intake such as whole grains and legumes, and avoiding late night snacks.

### Measurements of dietary adherence

Dietary adherence is divided into three categories: Poor: Not following the advice for more than five servings/day/week (non-adherent). Fair: Occasional deviation from following advice for 2–4 servings/day/week (adherent). Good: Strictly following diet advice for more than 5 servings/day/week (adherent).

### Healthy weight and anthropometric measurements

Participants will be classified according to WHO cutoff values for body mass index (BMI). Normal weight, overweight, obese and morbidly obese categories have a BMI of 18.5–24.9 kg/m^2^, 25–29.9 kg/m^2^, 30–34.9 kg/m^2^ and ≥ 35 kg/m^2^, respectively [41].

### Waist circumference

The waist circumference criteria used for diagnosis of abdominal obesity in the Arab and Middle Eastern population is ≥88 cm and ≥ 102 cm for women and men, respectively [37, 42].

### Behavioral change

As per the TTM [24], participants will be classified into five stages of changes (precontemplation (PC), contemplation (C), preparation, action, and maintenance) (Table 1) at the baseline visit and then every month during the first 6-month follow-up visit, through the WhatsApp application in the participants’ smartphones. This will test whether the sent messages were appropriate for the stage of behavioral change. This evaluation will be updated based on self-reported questions, which will be given to the participants separately and sent back to the research team through the WhatsApp application.

### Intervention

Interventions for this study will be on two levels. The standard health care, which is offered by primary health care services at King Saud University, Riyadh, Saudi Arabia and the text 4 change SMS messages.

### Message development

The content of the messages is derived from the WHO resources for a healthy diet, [31] physical activity and diabetes, Saudi National Diabetes Prevention and Control Program educational material [43], studies with similar interventions, online social listening, and clinical and personal experiences of Saudi culture [44]. The messages will be in formal Arabic with the infusion of some phrases and words from the common Saudi dialect. Messages are written in the second person, using you, your, and yours. Each stage of change of the TTM has a designated number of messages to promote a healthy diet and physical activity. The messages’ content was created according to the specific processes of change for each TTM stage. A total of 371 Arabic messages will be created by a health communication specialist and the project manager, who is well-acquainted with the target audience from a previous study. The specialist is well-acquainted with the Arabic speaking target population’s local accent, sentiments and culture. The messages’ content is based on international and national credible providers of health information and will be jointly prepared with a team of certified health educators. The messages will undergo cycles of feedback by a public health mental health specialist, a language reviser, and non-study participants from the target audience before the actual launch.

In the early stages of change (PC and C stages) that mainly require raising awareness, and contain nearly all essential messages needed to take the decision of change, every two messages are followed by a reminder, conveying the information mentioned in the previous messages every week (three for physical activity and three for a healthy diet). Reminders may take the form of text messages or multimedia messages (e.g. videos and infographics). For the following stages of change, where action is expected in the very near future or happening, the three weekly messages (for a healthy diet and physical activity) focus mainly on helping participants make a smooth transition through the process of change by providing a variety of messages conveying different alternatives of the same idea to achieve the goal of the stage.

The number of messages to cover 6 months are distributed as:

### Healthy diet messages

Healthy diet messages were 189 in total, distributed per stage as in precontemplation, contemplation, preparation, action and maintenance, 55, 48, 14, 60 and 12, respectively (Table 3).

**Table 3:** Number of messages according to the stage of change

Healthy diet messages according to the stage of change						
^a^Stages of change as per ^b^TTM	Pre-contemplation	Contemplation	Preparation	Action	Maintenance	Total
Number of messages	55	48	14	60	12	189
Physical activity messages according to the stage of change						
Number of messages	56	51	14	60	12	193
Total	111	99	28	120	24	382

^a^For each stage, three messages about diet and three messages about physical activity will be sent every week

^b^TTM, Transtheoretical model of change

### Physical activity messages

For physical activity, a total of **193** messages were created to cover the stages of change, namely precontemplation, contemplation, preparation, action, and maintenance, 56, 51, 14, 60 and 12, respectively (Table 3). The content covers the different processes of change (such as consciousness raising, helping relationships etc.) as suitable for each stage of change.

### Length and delivery method of the messages

The SMS phone messaging will be done through the King Khalid University Hospital commercial delivery manager website (Easy SMS manager) and sent to participants three times per week during working hours. Messages will most probably be sent in the morning between 10 and 11 am. On each assigned day, they will receive two messages (one for physical activity and the other for diet) according to their stage of change. The easy SMS manager allows a message length of 70 Arabic language characters. If this limit is exceeded, it will be counted as two messages instead of one, thereby increasing the cost. The system uses a standardized communication protocol assuring the delivery of text messages to the participants; however, receiving text messages from the participants or exchanging messages is not permitted (i.e. it is a one-way system) To ensure that messages were delivered, they will also be sent to one of the authors’ smartphones. At every visit, each participant will be asked about the number of messages received to ensure that all messages have been delivered and seen.

### Time of messaging

The messages will be sent to the intervention group only in the first 6 months from the baseline. The following time points measure the intervention’s long-term effect on biological markers, and the stages of change for healthy eating and physical activity.

### Measuring the TTM stage of change, the effect of intervention in the long term

The TTM stage of change for physical activity and a healthy diet is determined at the baseline using a question adjunct to the WHO STEPS questionnaire. At the baseline visit and every month, the adjunct questions will be delivered to the participants’ smartphone through the WhatsApp application. This will detect progress in the stage of change and help us move on to the next set of messages accordingly. The WHO STEPS questionnaire will be administered at every 6-month follow-up visit to measure the interventions’ long-term effects on biological markers and changes towards a healthy diet and physical activity.

### Acceptance of the text 4 change SMS

At the first 6-month follow-up visit, we will assess the interventional messages using a specially designed questionnaire (adjunct questions). It will assess the user’s acceptance and satisfaction of the SMS program, using a scoring system from 0 to 10. It will assess the messages’ content, frequency, clarity and personal view of the impact of messages on the participants’ lifestyle. The list of questions also includes the preferred time to receive messages, and invites suggestions for improvement. A total score of 10 denotes that the text messages were highly accepted and satisfactory, and zero denotes that text messages were the least accepted and satisfactory.

### Pilot study

Prior to commencing the actual study, we will recruit 30 non-study participants for a pilot study to be conducted in 1 month. We will assess the questionnaire for face validity, feasibility, and an estimate of the time taken to administer the questionnaire. Participants will be asked for verbal feedback while completing the survey. The SMS messages will be sent, based on the stage of change for each participant to review (from 408 messages) and give feedback on the ease of language and content. We will keep a feedback log to track all needed changes for the questionnaire and text messages. In this one-month pilot study, we will test the electronic messaging system, the feasibility of remote follow-up, and the documentation and data management strategy. Data collected in this pilot study will be analyzed and published.

### Study outcomes

By the end of the trial, we expect the following outcomes:

#### Primary outcome

Adoption of healthy lifestyle behaviors including healthy eating and physical activity practices complying with WHO recommendations. Incidence of T2DM, based on a change in HbA1c with an expected reduction of 1–2% at the end of the trial.

#### Secondary outcomes at 6-month follow-up visits

Change in the TTM towards stages of action and maintenance, decreased levels of LDL-C to < 130 mg/dl or ≤ 5.1 mmol/l, change in the systolic\diastolic blood pressure to normal blood pressure < 120/80 mmHg, respectively, decrease in body weight by 5–10% per year, improvement in the physical activity level to the recommended 150 min per week and improvement in the daily consumption of portions of fruit and vegetables to 5–7 servings/day.

### Data analysis plan

Data will be analyzed using the Statistical Package for Social Studies (SPSS 22; IBM Corp., New York, NY, USA). Continuous variables will be expressed as mean ± standard deviation or median and interquartile ranges according to the normality assessment. Categorical variables will be expressed as frequencies and percentages.

The chi-square statistical test will be applied for categorical variables to assess the association between them. The t-test or Mann-Whitney test will be used to compare numeric variables. Repeated measures analysis of variances or Friedman’s test will be used to evaluate repeated measures over the follow-up period.

The incidence rate and cumulative incidence rate will be computed for incidence of T2DM among the intervention and control groups. Additionally, comparison between the incidence of T2DM among different age groups and gender will be evaluated. Kaplan-Meier survival curves and log-rank tests will be used to compare the intervention and control groups as well as the different risk groups for progression to T2DM, and transition and/or relapses from different stages of TTM.

Hazards ratios with 95% confidence intervals will be calculated and the adjustment of known confounders will be adopted in the Cox regression model. In all the analyses, *P*-values < 0.05 will be considered statistically significant.

The principal analyses of primary and secondary outcomes will use the intent-to-treat approach. The intent-to-treat analyses will include all participants in their randomly assigned treatment groups regardless of a participant’s adherence to the assigned treatment regimen.

All data entry will be anonymous and identification numbers will be saved in a password-protected log file with the primary investigator. Backup of entered data will be created monthly to avoid any loss of collected data. An interim analysis of data will be conducted 12 months after recruitment of the last participants. Interim analysis aims to evaluate the completeness and accuracy of the collected data and early detection of any difficulties.

### Sample size calculation

A sample size of 300 is required so that there are 150 participants each in the intervention and control groups. The sample size was calculated by using Stata® software version 15 (StataCorp, College Station, Texas, USA) with the assumptions that α = level of significance (0.05), β = Type II error (power of the study = 1- β = 80%). The hazard rate of diabetes development among the control group is 10% over the 2 years, the hazard rate of diabetes development among the intervention group is 7% over the 2 years (reduction of about 30%) and the minimum sample size required to reject the null hypothesis is 256 (128 in each group). With an expected loss of follow-up around 20%, the sample size was increased to 300 participants in total.

### Ethical considerations and adherence to protocol

This study protocol was approved by the institutional review board (IRB) at King Saud University’s College of Medicine, approval number E-17-2607. It is also registered as an International Standard Randomized Control Trial, registration number ISRCTN10857643. All participants will sign a written consent form prior to enrolment in this trial at baseline. For the actual study participants, there will be non-monetary incentives of printed-out visual representations of their progress at follow-up visits.

For fidelity to the protocol, the project manager will oversee the workflow of the intervention, follow-up visits, managerial and data management components, and ensure compliance with the work plan. Furthermore, the IRB at King Saud University’s College of Medicine requires 6-monthly study progress reports, projects are monitored by random audits and require the submission of an annual report for protocol approval continuation.

To avoid possible bias, the laboratory staff, biostatistician, health educator, and principal investigator will be blinded to the participant’s group allocation until the end of the study. Neither the participants nor the research assistant/project manager responsible for disseminating the text messages will be blinded to the intervention due to its nature.

## Discussion

This trial aims to examine the effect of a TTM based on short messages (text 4 change) towards a lifestyle modification intervention to prevent or delay the onset of T2DM. The intervention will promote a healthy diet and increase in physical activity among subjects with IGT. We are targeting the eligible participants from the HHP study for this trial. The well-trained research team will deliver the intervention according to the TTM stages, to measure behavioral changes in the prediabetic participants. Therefore, the effectiveness of the SMS messages on behavioral change can be tested as proposed and planned.

This trial has been designed to address the scarcity in published studies about the effectiveness of lifestyle modification in preventing the occurrence of diabetes, using SMS as an intervention following the TTM model. Furthermore, the current trial will also provide evidence for progression and risk reduction in this category of participants with IFG or IGT. Applying this new approach to promote the importance of lifestyle modification in prediabetics is expected to delay and/or prevent the development of T2DM in Saudi Arabia. Subsequently, the risk of CV morbidity and mortality will also be reduced. Thus, results from this trial will promote an innovative and high-tech way to decrease the burden of CVD and diabetes management in Saudi Arabia.

## Data Availability

Not applicable.

## Abbreviations

ADA: American Diabetes Association
ATP-III: third adult treatment panel
BMI: Body mass index
C: Contemplation
CDC: Center for Disease Control and Prevention
CV: Cardiovascular
CVD: Cardiovascular diseases
HbA1c: Glycated hemoglobin
HDL-C: High-density lipoprotein cholesterol
HHP: Heart health promotion study
IFG: Impaired fasting glucose
IGT: Impaired glucose tolerance
IRB: Institutional review board
LDL-C: Low-density lipoprotein cholesterol
MetS: Metabolic syndrome
NCEP: National cholesterol education program
SMS: Short message service
T2DM: Type 2 diabetes mellitus
TTM: Transtheoretical model
US: the United States
WHO: World Health Organization
